# The fundamentals of eye tracking, Part 7: Determining data quality

**DOI:** 10.3758/s13428-026-03039-4

**Published:** 2026-06-02

**Authors:** Diederick C. Niehorster, Marcus Nyström, Roy S. Hessels, Jeroen S. Benjamins, Richard Andersson, Ignace T. C. Hooge

**Affiliations:** 1 Lund University Humanities Lab, Lund University, Lund, Sweden; 2 Department of Psychology, Lund University, Lund, Sweden; 3 Experimental Psychology, Helmholtz Institute, Utrecht University, Utrecht, The Netherlands; 4 Social, Health and Organizational Psychology, Utrecht University, Utrecht, The Netherlands; 5 Tobii AB, Danderyd, Sweden

**Keywords:** Eye tracking, Data quality, Accuracy, Precision, Data loss, Validation, Tools, Software

## Abstract

Understanding the quality of eye-tracking recordings, often characterized using accuracy, precision, and data loss, is crucial for the interpretation of eye tracking data. Eye-tracking data quality can furthermore place fundamental limits on what studies can be conducted with an eye tracker, and one may be required to report eye-tracking data quality when publishing a study. However, how does one determine the quality of eye-tracking data? This article provides an overview of operationalizations of accuracy, precision, and data loss and practical advice for determining eye-tracking data quality. Furthermore, the programming code for calculating various quality metrics for a segment of eye-tracking data is provided in MATLAB, Python, and R. Also provided is ETDQualitizer, a tool designed to enable anyone to easily determine the data quality of their recordings. We provide a version that is browser-based (https://dcnieho.github.io/ETDQualitizer) and enables determining eye-tracking data quality without installation or programming, while ensuring data privacy by running entirely locally. ETDQualitizer is further provided as a MATLAB, Python, and R library (https://github.com/dcnieho/ETDQualitizer) that can be integrated in one’s analysis scripts. We hope that this article enables any researcher to determine, critically evaluate, and report on eye-tracking data quality, and that it spurs researchers to adopt a data quality perspective in all their future eye-tracking studies.

## Introduction

This article is the seventh installment in a series on the fundamentals of eye tracking. The articles are aimed at individuals who are (one of) the first in their group, company, or research field to use eye tracking, with a focus on all the decisions one may make in the context of an eye-tracking study. Such individuals may come from academia (e.g., psychology, biology, medicine, educational science, computer science), commercial institutions (e.g., marketing research, usability, decision-making), and non-commercial institutions (e.g., hospitals, air traffic control, military organizations). Note that this is not an exhaustive description of the target audience. More experienced eye-tracking researchers may find useful insights in the article series, or may find the article series a useful reference or hub to relevant research. Previous papers in the series have dealt with (1) the relation between theory and research question (Hessels et al., 2024), (2) operationalizing research questions (Hooge et al., 2025), (3) choosing an eye tracker (Nyström et al., 2025a), (4) tools for eye tracking studies (Niehorster et al., 2025b), (5) the importance of piloting (Hessels et al., 2025b), and (6) working with AOIs (Hooge et al., 2026). One may either choose to start the series by reading the present article, but one may also first read earlier articles in the series. The present article addresses data quality in eye tracking research.

As previous parts of this article series have highlighted, constructing a research question that can be answered using eye tracking (Hessels et al., 2024), operationalizing the research question using appropriate eye tracking measures (Hooge et al., 2025) and choosing a suitable eye tracker with which to perform the recordings (Nyström et al., 2025a) can be a considerable endeavor. Experimental design texts may have one believe that once these hurdles have been taken, the hard part of conducting the study is now finished: thanks to a careful design everything across the study can be assumed to be equal except for one purposeful manipulation that the researcher introduced. This *ceteris paribus* assumption (Meehl, 1990; Lakatos, 1978) makes it possible for the researcher to safely ascribe any effects that they observe to the factor controlled in the researcher’s study, whether that be some manipulation between conditions, or a difference in some characteristic between participant groups. Experience tells us that research often is not that simple (see, e.g., Hessels et al., 2025b). Let us illustrate this point with several examples from our own research, in which it was critical to consider whether factors beyond the experimental manipulation could affect our conclusions. Specifically, we will examine studies in which considerations regarding eye-tracking data quality (e.g., offsets in the data or the amount of data loss) presented serious roadblocks or confounds, and discuss how we avoided these potential problems. Using these examples, we illustrate the importance of taking a data quality perspective and having insight into data quality when designing a study and interpreting the recorded eye-tracking data.

First, consider a study that examined eye contact during a collaborative task (Hessels et al., 2025a). In this study, eye contact was operationalized as looking at the partner’s face. One may wonder why we adopted such a coarse definition of eye contact and did not instead operationalize eye contact as looking at the other person’s eyes (Jongerius, Hessels, Romijn, Smets, & Hillen, 2020). We decided to use the whole face instead of only the eye region based on extensive pilot testing (described in Hessels et al., 2025b). This testing included examining what is feasible given the inaccuracy (the amount of offset from the true gaze direction) of the wearable eye trackers, and given the study’s setup where participants were placed at about 1.5 m from each other. These piloting efforts revealed that the inaccuracy of the wearable eye trackers (offsets of 1.73–3.37$^{\circ }$, Hessels et al., 2025a) was too large to support a more detailed analysis of face looking behavior (e.g., the distance between the eyes and mouth at 1.5 m would be less than 3 $^{\circ }$). A further consideration was that this study was a multi-site cross-cultural study involving a cohort of Dutch and a cohort of Japanese participants. Different eye trackers were used at the two sites, with different specifications for the quality of the eye-tracking data. Furthermore, data quality could be expected to differ between the two sites due to physical differences (e.g., nose size, which may lead to non-ideal positioning, or more movement, of the glasses) that relate to the ethnicity of the participants (e.g., Blignaut & Wium, 2014; Holmqvist & Andersson, 2017). This further motivated our choice to operationalize eye contact as looking anywhere on the face to minimize the possibility that any differences we report between the participant groups could be due to differences in accuracy instead of our object of study–differences in participants’ looking behavior. In summary, this example shows that knowledge about the inaccuracy of the eye tracking recordings is critical for determining the appropriate spatial scale at which the data is analyzed, e.g., determining the size of areas of interest (AOIs, Hooge et al., 2026; Hessels, Kemner, van den Boomen, & Hooge, 2016a).

**Fig. 1:**
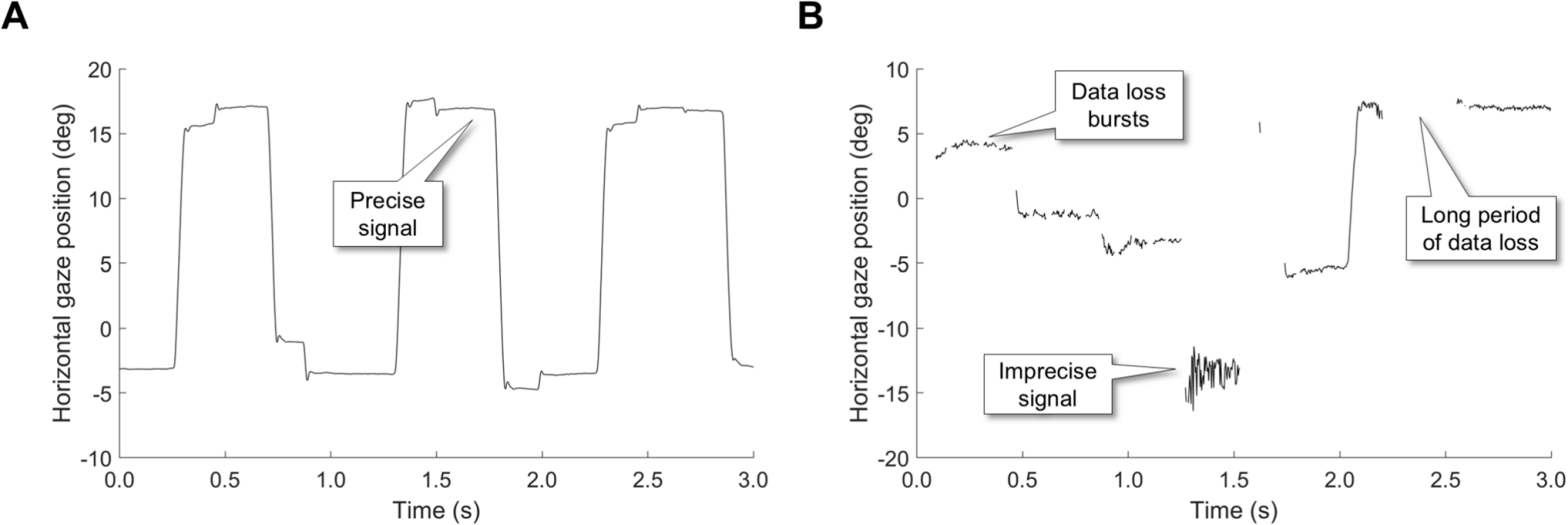
Eye-tracking data quality. **A** Horizontal gaze position of the right eye as a function of time, recorded from an adult participant making saccades between two points on a screen with an EyeLink 1000 by Hooge, Nyström, Cornelissen, and Holmqvist (2015). **B** Horizontal gaze position of the right eye as a function of time, recorded from an infant participant viewing a search display with the Tobii TX300 by Hessels, Hooge, and Kemner (2016b). *Call-outs* indicate the relatively precise (very flat) gaze-position signal in panel A, and in contrast the imprecise (more variable) gaze-position signal in panel B, as well as short and long gaps in the gaze-position signal (data loss) in panel B. Note that the vertical axis range is the same between the two panels, making the figures directly comparable

Second, consider a study into the looking behavior of children of different ages (e.g., Jones & Klin, 2013; Hunnius, Geuze, & van Geert, 2006; Lewkowicz & Hansen-Tift, 2012). When comparing the looking behavior of groups of children of different ages (e.g., 5 months, 10 months, 3 years, and 9 years old, Hessels, & Hooge, 2019), suppose that one finds that fixation durations become shorter with age. Since the children were shown the exact same stimuli, were recorded with the same eye tracker, and the data were analyzed with the same procedure, is it now safe to conclude that some form of cognitive development has taken place, reflected in a decrease in fixation durations? We would argue that this cannot be safely concluded because at least one important potential confound has been overlooked: data quality. The quality of eye tracking data recorded from 5-month-old children is very different from that of 10-month-old children, and let alone that of older children or adults (see Fig. 1; Hessels & Hooge, 2019; Dalrymple, Manner, Harmelink, Teska, & Elison, 2018; Wass, Forssman, & Leppänen, 2014; Hooge, Niehorster, Nyström, Andersson, & Hessels, 2018; Hessels, Andersson, Hooge, Nyström, & Kemner, 2015a). The data for young children are often less precise and contain more data loss. This may be due to several factors, including differences in ease of positioning young children in front of the eye tracker, the ability to instruct the participant, behavioral differences during the recording, such as head movement, etc. Can such differences in precision and data loss instead explain the change in observed fixation durations? Since many of the analysis procedures often used for eye tracking data are not built for data of low quality, the estimated fixations and fixation durations may differ tremendously when data quality improves with age (Shic, Chawarska, & Scassellati, 2008; Wass et al., 2014; Hessels, Niehorster, Kemner, & Hooge, 2017). As such, observed differences in fixation duration may not be due to any change in the participant’s looking behavior, but instead only because of differences in data quality, which is clearly undesirable. For this reason, special fixation classification algorithms have been developed (e.g., Wass, Smith, & Johnson, 2013; Hessels et al., 2017; van Renswoude et al., 2018) with the explicit aim to be robust to differences in precision and data loss, which are now commonly used in developmental eye-tracking studies. This example shows the importance of ruling out differences in imprecision or data loss as a driving factor in differences in eye-tracking metrics between conditions or participant groups, and of being aware of these data quality aspects when choosing appropriate analysis methods.

Third, we provide an example in which eye-tracking data quality posed a potential roadblock to conducting a study. In Hooge et al. (2024a), we were interested in how accurately participants are able to refixate a target. It was clear that the measurement capabilities of the video-based eye trackers we had easy access to in our lab were insufficient for this study. These eye trackers offer an accuracy of only about 0.5 $^{\circ }$ with significant fixation-to-fixation variability, while we expected that humans would be able to refixate targets much more consistently than that. To exclude the possibility that the eye tracker is the limiting factor in the refixation accuracy, we had to look elsewhere for a more accurate eye tracker to make it possible for us to carry out our study. This better machine was found in the form of a retinal eye tracker (Bartuzel et al., 2020), which offers accuracy that is far superior to that of conventional video-based eye trackers (see Hooge et al., 2024a, for details), and, importantly, good enough for our investigation. This example shows the importance of considering the magnitude of expected errors in the recorded eye tracking data when determining whether an eye tracker is suitable for conducting the planned study.

The above three examples illustrate the importance of considering eye-tracking data quality in designing, analyzing, and reporting a study. Indeed, McConkie (1981) already argued that considering data quality is critical when conducting eye tracking studies, “In order to judge the degree of confidence one should have in the results of an experiment using eye movement records as data, it is necessary to have information about the quality of the eye movement data themselves.” (p. 97) His statement highlights that empirical scientists are limited in what they can conclude by the quality of their data, and should thus be aware of the data quality of their recordings when analyzing their data and formulating their conclusions. However, are avoiding confounds and ensuring that the eye tracker signal has suitable specifications for the planned study (cf. Nyström et al., 2025a) the only reasons a researcher may wish to take an interest in the data quality of their eye tracking recordings? No, at least three further reasons can be thought of. First, not only the researchers conducting the study but also critical readers (e.g., colleagues, reviewers) need sufficient information about the quality of the study’s eye-tracking recordings to decide whether the study’s conclusions are adequately supported by the collected data. In his article, McConkie (1981) therefore calls for authors to report the quality of their eye tracking data in their articles (see also, e.g., Holmqvist, Nyström, & Mulvey, 2012; Orquin & Holmqvist, 2018; Dunn et al., 2023). Second, one may wish to use information about the data quality of eye tracking recordings for data selection, removing recordings or trials that do not meet the minimum bar for inclusion in the study’s analysis (e.g., Hessels et al., 2022; Hessels et al., 2023; Sharafi et al., 2020; Masulli et al., 2022). Third, being able to determine the data quality of a given eye tracking setup is instrumental in being able to improve the quality of the setup, since it provides the information that is needed to determine if data quality is sufficient and the feedback required to judge whether changes to the setup or experiment improve the situation. It is important to realize that the data quality of a specific eye tracker is not a set of fixed values that are set in stone on the specification sheet. Instead, the actual performance a researcher can expect from their eye tracker critically also depends on, for instance, the setup and task (table, chair, lighting conditions, participant movement, see, e.g., Hessels, Cornelissen, Kemner, & Hooge, 2015b; Niehorster, Cornelissen, Holmqvist, Hooge, & Hessels, 2018; Niehorster et al., 2020c; Niehorster et al., 2025b; Hooge, Niehorster, Hessels, Cleveland, & Nyström, 2021; Hooge, Niehorster, Hessels, Benjamins, & Nyström, 2022) and participant characteristics (see, e.g., Nyström, Andersson, Holmqvist, & van de Weijer, 2013; Hessels et al., 2015a). As such, data quality can be influenced by the researcher through changes to, for instance, their setup, task, and instructions, and even their analysis methods (see Hessels et al., 2025b, for an in-depth discussion).

The above three examples also illustrate that there are no absolute standards for what “good” or “bad” eye tracking data is. As McConkie (1981) already said, “it is not appropriate to adopt standards concerning what is acceptable data; that varies with the nature of the questions being studied.” (p. 103) Indeed, the examples above illustrate that whether data quality is “good enough”, i.e., sufficient to be able to execute a study, depends on the study’s context, including the question being asked, the stimuli, the behavior of interest, and the analysis methods. This realization only further underscores the need for a researcher to be aware of the data quality of their eye-tracking recordings when designing, analyzing, and reporting a study.

In summary, there are many reasons why a researcher may (and, we would argue, *should*) take an interest in the quality of eye tracking data. However, how does one operationalize, determine, and report on the quality of eye tracking data? That is what this article deals with. In the rest of this article, we will discuss three common data quality concepts that are important to most studies using eye trackers: accuracy, precision, and data loss. In this article we aim to give a self-contained discussion of these three aspects of eye-tracking data quality, aiming to make the reader comfortable with these concepts and providing them with the knowledge and tools needed to determine, interpret and report accuracy, precision and data loss, as well as to reason about how these aspects of eye tracker data quality may affect their study^1^. Specifically, we will first discuss the concepts of accuracy, precision, and data loss and provide common operationalizations of each. We also provide algorithmic implementations of the operationalizations of the measures in MATLAB, Python, and R. We then cover how eye-tracking data quality metrics can be determined in practice. To this end, we provide a procedure for acquiring the data required for determining data quality, and a set of self-contained tools that can determine the data quality of recorded eye-tracking data. Finally, we provide guidance on reporting eye-tracking data quality in a scientific article. It should be noted that, with the exception of the new tools presented in this paper, the content of this article is relevant to all branches of eye tracking, regardless of the type of eye tracker used (e.g., screen-based, head-worn, or integrated in a VR, AR or XR headset), the eye tracking technology used (e.g., video-based using classical or deep learning methods, electro-oculography, or retinal tracking) or application (research, clinical or commercial use such as usability). We think that adopting a data quality perspective in the design, execution, and reporting of a study will raise the quality of eye-tracking research, regardless of the specific use one has for eye tracking.

## Operationalizations of eye tracking data quality

Before we delve into a discussion of the accuracy, precision, and data loss of eye-tracking data, we wish to make a point about the terminology used in this paper. While accuracy and precision are the officially sanctioned terms (see BIPM et al., 2012), we think that they are unwieldy to use in practice since they do not align with intuition. Consider the following example sentence: *according to the specifications of the manufacturer, the accuracy of the Pupil Neon is higher (1.8* $^{\circ }$*) than that of the Pupil Invisible (4.6* $^{\circ }$*)*. In this sentence, “higher” refers to the concept of accuracy itself (higher is better) and not to the value associated with the operationalization of accuracy (an offset; lower is better). That higher (or better) accuracy is represented by lower values appears counterintuitive, and the extra mental step required to correctly interpret sentences such as the above impedes understanding. The same problem exists for precision, where higher/better precision is also indicated by lower values. For this reason, in this manuscript, we will mostly use the terms inaccuracy and imprecision. Using this terminology, both interpretations are aligned, to wit: *the inaccuracy of the Pupil Neon is lower (1.8* $^{\circ }$*) than that of the Pupil Invisible (4.6* $^{\circ }$*)*.

In the rest of this section, we will discuss inaccuracy, imprecision, and data loss. For each of these eye-tracking data quality concepts, we first offer a formal definition and a conceptual discussion of what aspect of the eye-tracking signal it aims to assess. We then provide common operationalizations for quantifying these data quality aspects in eye-tracking signals. For these operationalizations, a conceptual explanation, a graphical representation, a mathematical representation, and an algorithmic implementation are provided. Here we wish to emphasize that we do not expect all readers to read every word in this section. Readers who prefer mathematical or code representations may opt to read this section start to finish. Other readers who want to only understand the concepts sufficiently to be able to use them in practice but are not mathematically or programming-inclined may, however, choose to skip the math and code and only read the conceptual explanations and examine the graphical representations. In the next section, these readers will find ready-made tools for determining data quality for their eye-tracking data, which are implemented using the discussed measures. As such, using the measures presented in this article only requires a grasp of what they represent, but not a detailed mathematical understanding, nor programming affinity.

The algorithmic implementations of each operationalization of a data quality measure are provided in the form of self-contained functions in  ,  , and  (this color coding will be used throughout the paper to identify the language in which a code snippet is written) that one can use directly to calculate a specific eye-tracking data quality measure for a segment of eye tracking data. These three languages were chosen because a recent overview of eye-tracking tools, Niehorster et al. (2025b), showed that they are the most common environments for eye-tracking data analysis. To be able to follow the mathematical and algorithmic implementation parts of this section, one needs to be familiar with some general terms regarding the format and coordinate system in which the gaze data is delivered by the eye tracker, such as gaze positions on a screen expressed in pixels and angular orientations of an eye expressed in degrees. One may also need to be able to transform gaze data from one representation or coordinate system to another. The reader is referred to Appendix A for a step-by-step pedagogical walk-through of these topics, should they wish to brush up their understanding.

How are inaccuracy, imprecision, and data loss defined? These are concepts that are not specific to eye tracking, but are used far more widely to characterize data acquired by measurement devices. As such, let us turn to the International Vocabulary of Metrology (BIPM et al., 2012) and adapt its definitions to the case of eye tracking recordings. *Accuracy* is defined as “the closeness of the gaze position reported by the eye-tracking setup to the actual gaze position of the participant” (Niehorster et al., 2020c). *Precision* “is defined as the closeness of a set of repeated gaze position measurements obtained under identical conditions (i.e., obtained from an eye that has not rotated)” (Niehorster et al., 2020d). Finally, *data loss* “is the relation between the number of measurements achieved by an eye-tracking setup in a given time interval and the number of measurements that should be expected based on the specifications of the eye-tracking setup” (Niehorster et al., 2020c; see also Hooge et al., 2022). Let us explore each of these in more detail.

### Inaccuracy

**Fig. 2:**
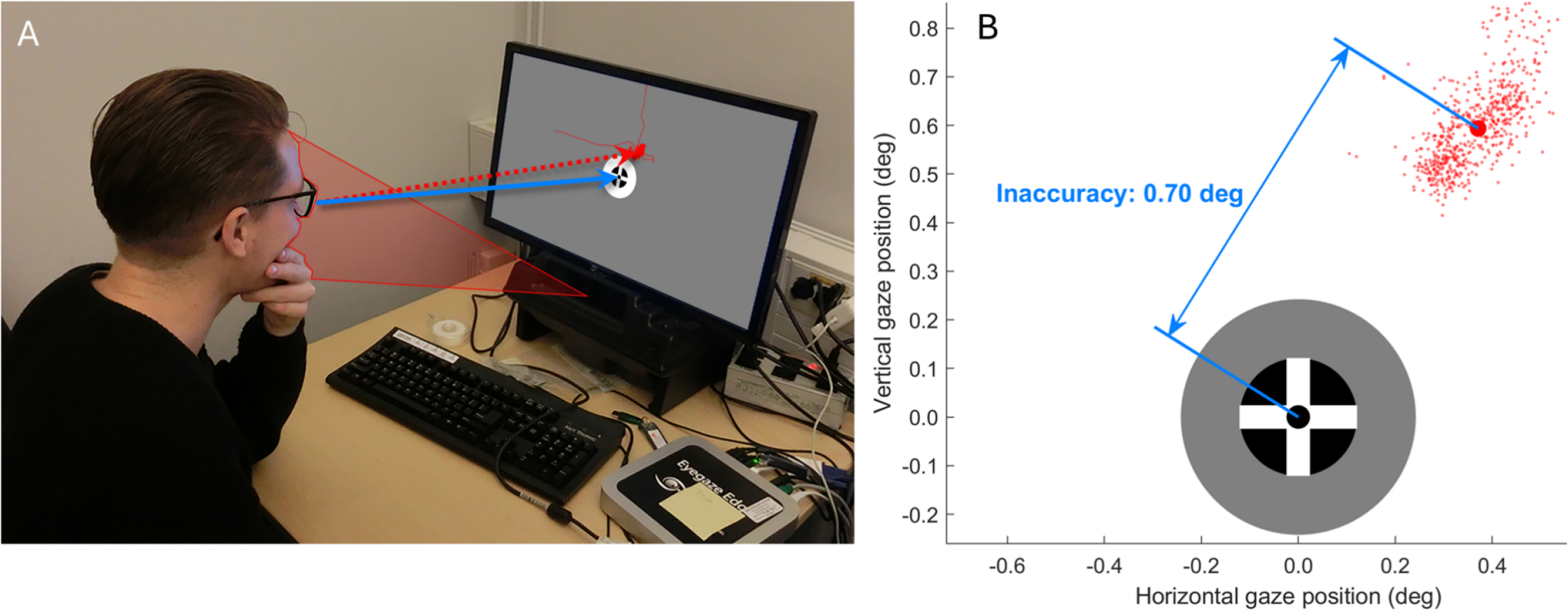
Inaccuracy. **A** A participant is instructed to look at a fixation target placed at the center of a screen attached to an eye tracker (*blue arrow*). The gaze data recorded by the eye tracker while the participant looks at the fixation target, as well as data recorded prior to this fixation, is indicated in *red* on the screen. The mean gaze position reported by the eye tracker for the episode where the participant looked at the fixation target is indicated by the *red arrow*. **B** Close up of the fixation target and 1 s of recorded gaze data (each *small red dot* is a gaze sample) on the eye tracker’s screen. Using such data, the inaccuracy can be quantified as the distance (*blue arrow*) between the center of the fixation target and the mean gaze position reported by the eye tracker (*red dot*). The inaccuracy in this example is 0.70 $^{\circ }$

Inaccuracy can be thought of as an offset in the recorded gaze positions from a “true” (or actual) gaze position. For instance, the gaze positions recorded by an eye tracker while a participant is instructed to fixate the center of a fixation target may show a consistent offset from the fixation target (Fig. 2).

Inaccuracy is often determined by means of an accuracy assessment (in the field of eye tracking, such an assessment is often referred to as a “validation”). Such accuracy assessment is often performed directly after calibrating the eye tracker^2^, but can also be performed at other moments during the recording. Since the inaccuracy of a gaze position measurement is defined as the offset between the measured gaze position and a true gaze position, some way of knowing the participant’s true gaze position is required to be able to assess accuracy. Therefore, one way accuracy assessments are commonly done is to present a fixation target to the participant, and *assume* that the participant accurately fixates the target. Well-behaved healthy participants can fixate targets very accurately (e.g., Hooge et al., 2024a; Poletti & Rucci, 2016). However, in our experience, participants new to eye tracking nonetheless may need a few runs through an accuracy assessment procedure before they display the desired fixation behavior. They may, for instance, look away from a fixation target too soon or be distracted (Harezlak, Kasprowski, & Stasch, 2014). For other populations, such as participants with oculomotor disorders, the assumption that the participant accurately fixates the target may not hold (e.g., Dunn et al., 2019; Rosengren, Nyström, Hammar, & Stridh, 2020). Automated procedures for assessing inaccuracy (and likewise for calibrating an eye tracker, which also relies on the assumption that the participant accurately fixates the target and often uses similar procedures as validation) may fail if the participant does not look where the procedure assumes they do and the procedure is not able to detect the participant’s failure to follow the instructions (Niehorster et al., 2024; Hessels et al., 2015a).

Typically, validation is performed using one or more locations or objects in the world such as fixation targets on computer screens (Fig. 2A, see, e.g., McConkie, 1981; Holmqvist et al., 2011; Niehorster and Nyström, 2020; Niehorster, Andersson, & Nyström, 2020a) or physical targets placed in the world (e.g., Fu et al., 2024; Niehorster, Hessels, Benjamins, Nyström, & Hooge, 2023; Babcock & Pelz, 2004; Evans, Jacobs, Tarduno, & Pelz, 2012) that participants are explicitly instructed to fixate. However, validation can also be performed using moments in a recording where the true gaze position of a participant can be inferred with reasonable certainty from the content of the visual stimulus (e.g., sparse stimuli are used where the target looked at can be ascertained with reasonable certainty, see, e.g., Špakov, Istance, Hyrskykari, Siirtola, & Räihä, 2019) or from the participant’s behavior (e.g., participants tend to look on elements of a user interface that they interact with through mouse clicks, Hornof & Halverson, 2002; Blignaut, Holmqvist, Nyström, & Dewhurst, 2014).

Is the inaccuracy of the recorded gaze data constant over the course of a recording? No, the gaze position reported by the eye tracker may vary between fixations of the same target and may even change (drift) during a single fixation. While these variations may in part be ascribed due to inaccurate fixation and small changes in eye position during fixation (McConkie, 1981; Rolfs, 2009), there are good reasons to believe that these changes in reported gaze position are at least in part due to the eye tracker. Indeed, the gaze position signal reported by most eye trackers not only reflects eye orientation, but is also affected by other factors. Several factors that may affect the gaze position reported by the eye tracker in the absence of eye rotation are discussed by McConkie (1981). For instance, a change in the position of the eyes with respect to the eye tracker (e.g., due to head movement on a chinrest, or slippage of head-worn eye trackers on the participant’s face) can yield significant changes in recorded gaze position (Niehorster et al., 2020c; Hessels et al., 2015b; Niehorster et al., 2018; Špakov, Isokoski, & Majaranta, 2014; Hermens, 2015; Cerrolaza, Villanueva, & Cabeza, 2012; Merchant, Morrissette, & Porterfield, 1974; Houben, Goumans, & van der Steen, 2006; Kolakowski & Pelz, 2006; De Reus, Zon, & Ouwerkerk, 2012). Furthermore, changes in pupil size may lead to apparent gaze shifts in the absence of changes in gaze position in many commonly used eye trackers (Wyatt, 2010; Drewes, Masson, & Montagnini, 2012; Drewes, Zhu, Hu, & Hu, 2014; Choe, Blake, & Lee, 2016; Hooge et al., 2021). Indeed, Hooge et al. (2024a) have argued that this pupil size artifact (PSA) presents a fundamental limit to the accuracy of current video-based eye trackers.

Given a segment of gaze position data, how does one compute the inaccuracy? A general way to operationalize inaccuracy is as the angle between the gaze vector and the vector from the eye to the center of the fixation target. The angular offset between the fixation target and the reported gaze direction can be computed using the inner product between the two vectors:

1 $$\begin{aligned} \text {inaccuracy} = \arccos \Big (\frac{\vec {v}_{\text {gaze}}}{\Vert \vec {v}_{\text {gaze}}\Vert } \cdot \frac{\vec {v}_{\text {target}}}{\Vert \vec {v}_{\text {target}}\Vert }\Big ) \end{aligned}$$

where $\vec {v}_{gaze}$ is the gaze vector (red arrow in Fig. 2A), $\vec {v}_{target}$ the vector connecting the eye and the fixation target (blue arrow in Fig. 2A), $\Vert \vec {v}\Vert$ denotes the length of the vector, the dot ($\cdot$) denotes the inner (dot) product of the vectors and $\text {arccos}()$ is the inverse cosine function.

Equation 1 is used to calculate the angular offset of a single gaze position to a target. How does one then compute the offset of a whole segment of gaze data recorded while a participant looks at a fixation target to the center of this fixation target? For this, one first has to select the samples to include in the calculation, for instance by determining which samples were recorded during a fixation on the target. Several strategies can be used for sample selection, such as first performing fixation classification on the segment of gaze data and then selecting the longest or the closest fixation, selecting a window of minimum dispersion or relying on knowledge about when the fixation target was displayed to the participant (Niehorster, Zemblys, & Holmqvist, 2021; Niehorster et al., 2020a; Niehorster et al., 2023; Chernyak, Chernyak, Bland, & Rahier, 2021; Nyström et al., 2013; Dalrymple et al., 2018). Second, once the gaze position samples for the accuracy calculation have been selected, a single inaccuracy should be determined for the whole segment. This can be done by first computing an average gaze vector using, for example, the mean or median of the selected gaze positions, and then plugging this average gaze vector into Eq. 1.

The function |accuracy()|^3^ (Listings 1–3) implement Eq. 1 for a sequence of gaze positions and a single fixation target. Input arguments target_azi and target_ele specify the target direction, and azi and ele represent a segment of gaze directions, both expressed as azimuth and elevation angles (see the “Expressing gaze data as positions, angles or vectors” appendix). Internally, the gaze directions are first converted to gaze vectors, which are then processed into a single representative gaze direction by applying the operation specified by central_tendency_fun (by default, the mean). The offset of this gaze direction from the target direction is then determined. The output of the code snippets is the angular offset of gaze from the target, in degrees.
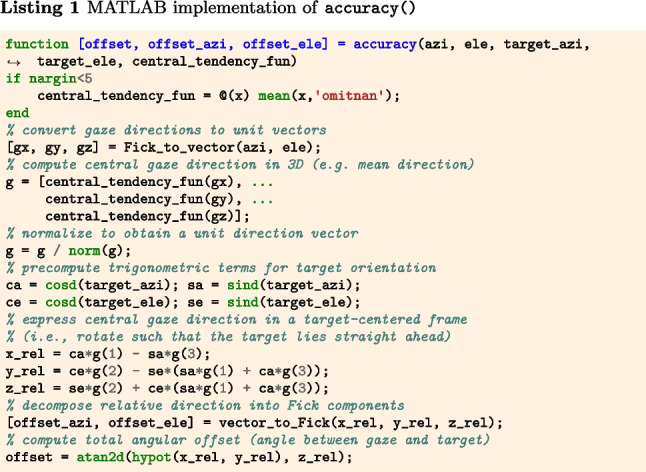

**Fig. 3:**
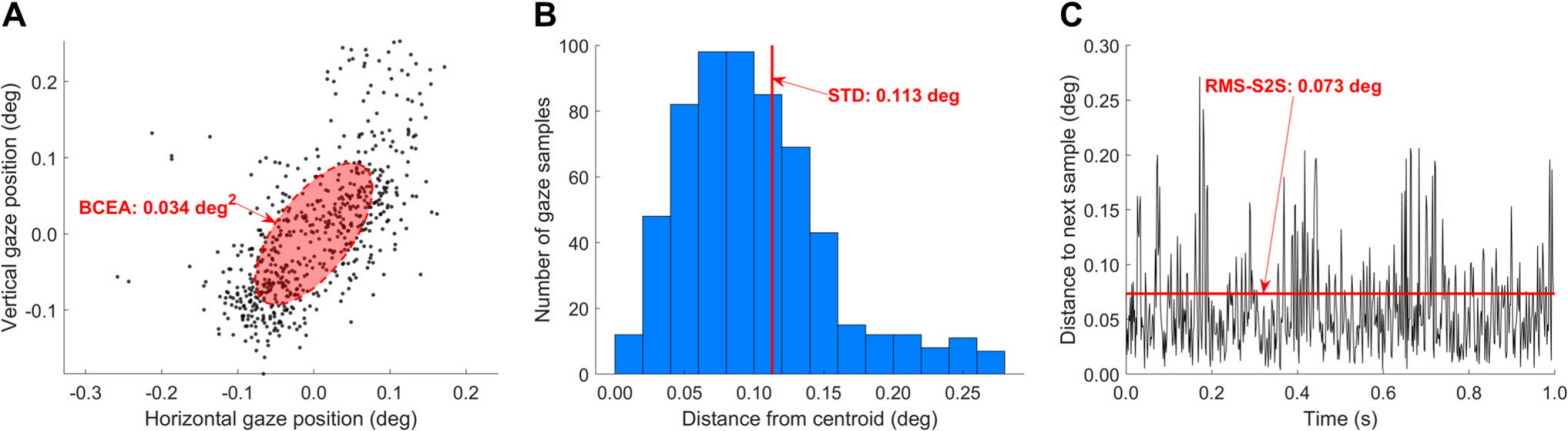
Imprecision. **A** To compute the BCEA, an ellipse is fit to a (1 s) segment of recorded gaze (each *small black dot* is a gaze sample). In this case, $P=0.68$ was used for fitting the ellipse, which means that approximately 68% of the gaze samples is contained within the ellipse. **B** A visualization of the STD precision measure. Shown is the distribution of distances of the gaze samples from their centroid. The red line indicates the corresponding standard deviation precision measure. **C**. A visualization of the RMS-S2S precision measure. Shown is the distance between adjacent gaze samples over time for the 1 s segment of gaze data, along with the corresponding RMS-S2S value (indicated by the red line)

Finally, the above discussion considered accuracy assessment using a single fixation target. The accuracy of the eye tracker may, however, vary over the stimulus space (e.g., Schlegelmilch & Wertz, 2019; Niehorster et al., 2020c; Popelka, Stachoň, Sašinka, & Doležalová, 2016). As such, during accuracy assessment, often multiple gaze targets are presented to the participant, for instance spanning the range of the visual stimulation used during the recording (Holmqvist et al., 2011; Niehorster et al., 2020a, 2023; O’Regan, 1978; McConkie, 1981). Such procedures yield multiple inaccuracy values, one per fixation target. As is appropriate for the design of the study, overall inaccuracy can then, for instance, be determined as the mean or median inaccuracy over all fixation targets, be determined as the mean or median of targets at a certain eccentricity, or inaccuracies can be directly reported for individual fixation targets. 
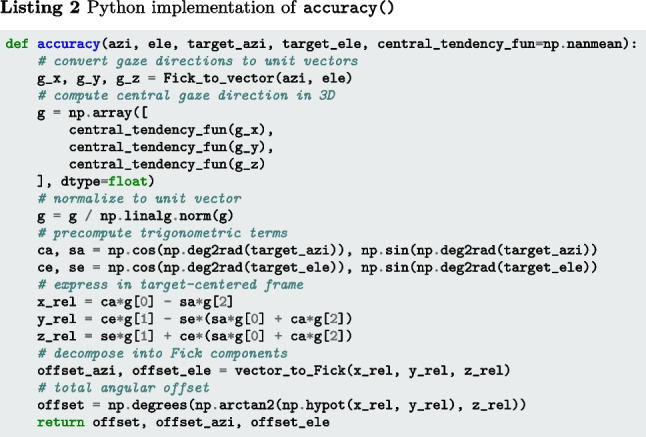

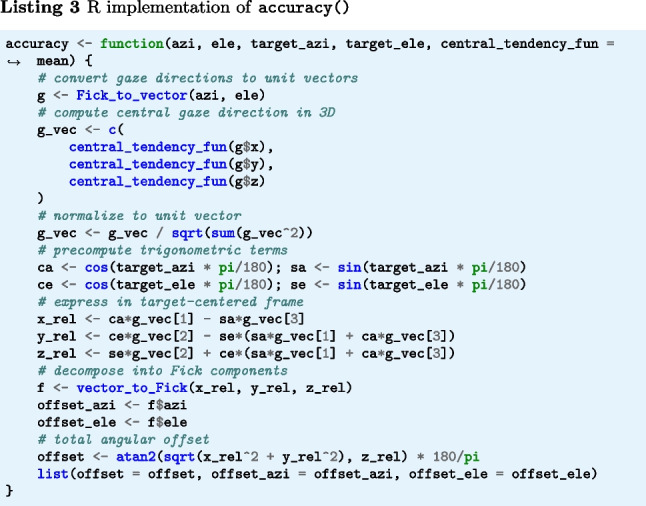


### Imprecision

Imprecision can be thought of as the magnitude of variability in an eye tracker signal. As can be seen in Fig. 3, even when the eye is presumably steadily fixating, the gaze position signal reported by the eye tracker contains variation, exhibited both as a spatial spread of the recorded gaze positions (Fig. 3A) and as fluctuations in recorded position from one gaze position sample to the next (Fig. 3C). This variation in the eye tracking signal during fixation, sometimes referred to as “noise”, is likely due to both measurement error in the eye tracker and due to small eye movements made during fixation (Rolfs, 2009; Niehorster et al., 2021). However, unless a high-end eye tracker with a very low noise level is used, the fixational eye movements are likely (very) small compared to the magnitude of the measurement noise of the eye tracker (McConkie, 1981).

The imprecision of the eye tracking signal may provide an indication of the smallest change in gaze direction that can be measured with the device. This is intuitive: the larger the variability in the eye tracking signal, the larger changes (eye movements) in the signal need to be, for it to be possible to reliably detect these changes. However, it should be recognized that this is not the whole story. The detail that can be resolved can also be improved, even in the face of high imprecision, by either averaging over longer episodes of eye tracking data, or by averaging together a larger number of eye movements, such as saccades (e.g., Hooge, Nyström, Cornelissen, & Holmqvist, 2015; Nyström et al., 2025b), or eye tracking episodes (e.g., Allopenna, Magnuson, & Tanenhaus, 1998).

There are many aspects of imprecision of a gaze position signal that can be examined (Niehorster et al., 2020d; Holmqvist & Andersson, 2017). Here we consider two aspects that underlie commonly used measures of the imprecision of an eye tracking signal: the spatial spread in a recorded eye tracking signal (Fig. 3A and B), and sample-to-sample fluctuations (Fig. 3C). As discussed previously (e.g., Niehorster et al., 2020d; Blignaut & Beelders, 2012), these quantify different aspects of the imprecision of a signal. Intuitively, as detailed in Niehorster et al. (2020d), the former group of measures provides an indication of the spatial stability of the signal, whereas the latter provides an indication of the speed of the signal. It should be noted that sample-to-sample fluctuation measures can be applied not only to the eye tracker’s gaze position signal, but to any signal provided by the eye tracker, such as the pupil size or eye openness (Nyström, Andersson, Niehorster, Hessels, & Hooge, 2024).

Next, we introduce a measure that assesses the magnitude of sample-to-sample fluctuations in a signal (RMS-S2S) and two measures of the spatial spread of a signal (STD and BCEA).

#### RMS of sample-to-sample deviations (RMS-S2S)

RMS refers to the root mean square of the displacement between successive samples in a gaze signal (Fig. 3C). While often simply referred to as RMS, RMS-S2S is the more precise term. RMS-S2S is calculated by the following formula (Niehorster et al., 2020d):

2 $$\begin{aligned} \begin{aligned} \text {RMS-S2S}&= \sqrt{\overline{({\theta _i}^2)_{i=1}^{n-1}}}\\&= \sqrt{\frac{1}{n-1} \sum _{i=1}^{n-1} {\theta _i}^2}\\&= \sqrt{\frac{1}{n-1} \sum _{i=1}^{n-1} \Big ((x_{i+1} - x_i)^2 + (y_{i+1} - y_i)^2\Big )} \end{aligned} \end{aligned}$$

where *n* is the number of samples in the gaze signal for which the RMS-S2S value is to be calculated, *x* and *y* the horizontal and vertical components of the gaze signal, $\theta$ the distance between two successive gaze positions, $({\theta _i}^2)_{i=1}^{n-1}$ the sequence of squared intersample distances for *n* gaze samples and $\overline{({\theta _i}^2)_{i=1}^{n-1}}$ the mean of this sequence.

Since the goal of RMS-S2S is to operationalize the variability in the gaze signal due to the eye tracker in the absence of physical eye movement, RMS-S2S is typically implemented by estimating the noise level during fixations when actual eye movement is presumed to be minimal. Indeed, Eq. 2 is appropriate for calculating the variability in a gaze signal obtained during fixation, but not during periods of significant change in gaze position, such as during saccades, pursuit, or VOR. For instance, the large displacements during a saccade would bias the calculated RMS-S2S value towards a larger value. It has furthermore been shown that the RMS-S2S measure is very sensitive to spikes (large but very brief excursions) in the gaze position signal (e.g., Blignaut & Beelders, 2012; Niehorster et al., 2020d). Alternative formulations and implementations of the RMS-S2S measure have been proposed that do not suffer from these problems and can be used to estimate the RMS-S2S precision for longer segments of gaze data containing fixations, saccades, and occasional extreme spikes. First, some authors (e.g., Niehorster et al., 2023; Niehorster, Hessels, & Benjamins, 2020b; Hessels et al., 2023; Hooge et al., 2018; Hooge, Niehorster, Nyström, & Hessels, 2024b) compute RMS-S2S using a sliding window technique. In this technique, Eq. 2 is used to compute RMS-S2S in a window (e.g., 200 ms long) that is slid over the data. The RMS-S2S for the entire gaze signal is then determined as the median RMS-S2S across all windows. Since it is reasonable to assume that saccades make up (way) less than 50 % of the signal, the thus-derived RMS-S2S value provides an estimate of the precision during fixations. Second, other authors (e.g., McConkie, 1981) simply adapt Eq. 2 to use the median instead of the mean squared sample-to-sample distance:

3 $$\begin{aligned} \begin{aligned} \text {RMS-S2S}&= \sqrt{{{\,\textrm{med}\,}}\Big (({\theta _i}^2)_{i=1}^{n-1}\Big )} \end{aligned} \end{aligned}$$

where ${{\,\textrm{med}\,}}()$ refers to the median operation.

The below function rms_s2s() (Listings 4–6) implements Eqs. 2 and 3. The input arguments azi and ele are segments of gaze directions expressed as azimuth and elevation angles in degrees. The output is RMS-S2S precision in degrees.
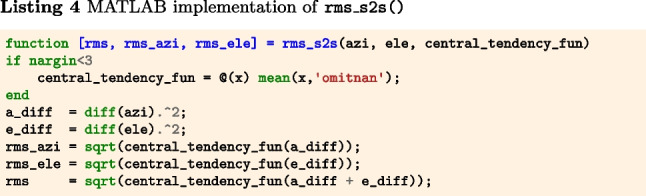

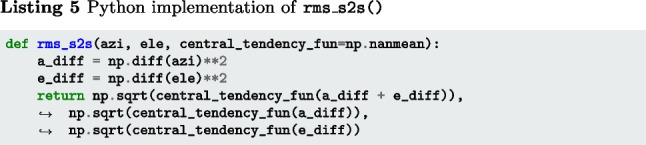

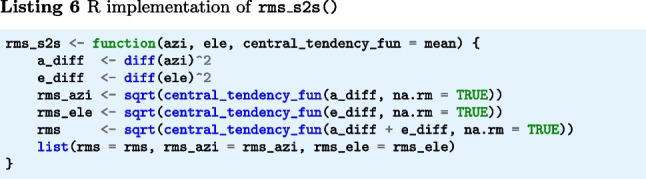

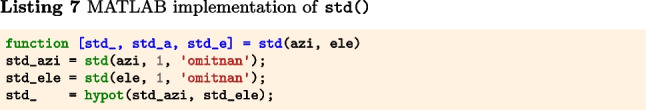

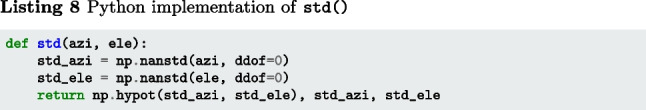

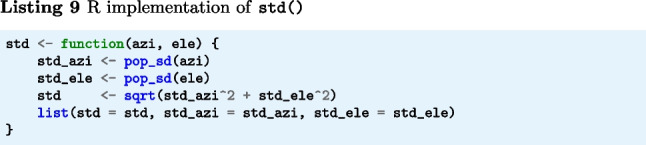

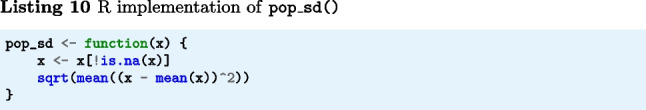


#### Standard deviation (STD)

Standard deviation is a measure of the spatial spread of a segment of gaze position samples (Fig. 3B), and is calculated as follows:

4 $$\begin{aligned} \begin{aligned} \text {STD}&= \sqrt{\frac{1}{n} \sum _{i=1}^n \Big ((x_i - \overline{x})^2 + (y_i - \overline{y})^2\Big )}\\&= \sqrt{\text {STD}_x^2+\text {STD}_y^2} \end{aligned} \end{aligned}$$

where *x* and *y* are the horizontal and vertical components of the gaze signal, and $\overline{x}$ and $\overline{y}$ denote the mean horizontal and vertical gaze position of the segment. Note that the STD calculated in this manner is a one-dimensional measure that characterizes the radial extent of the gaze position segment.

The function std() (Listings 7–9) implements Eq. 4. The input arguments azi and ele are segments of gaze directions expressed as azimuth and elevation angles in degrees. The output is STD precision in degrees.

Note that this R function requires a custom function for calculating the population standard deviation (Listing 10).
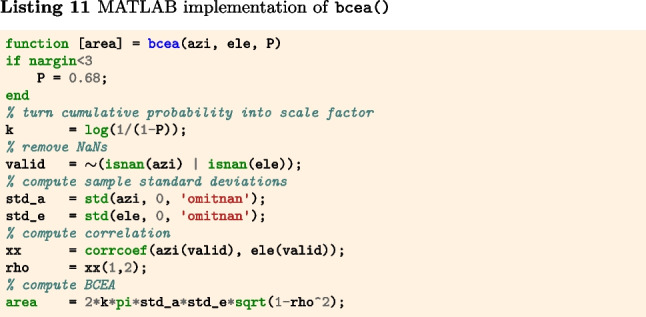


#### Bivariate contour ellipse area (BCEA)

In contrast to STD, the bivariate contour ellipse area (BCEA) is a two-dimensional (area) measure for characterizing the spatial spread of a segment of gaze position data (Fig. 3A). This measure is often used in the clinical literature as a measure of fixational instability (e.g., Crossland & Rubin, 2002; Steinman, 1965; Chung, Kumar, Li, & Levi, 2015; Timberlake et al., 2005). The BCEA value represents the area of an ellipse that encompasses a certain percentage of the gaze positions in the segment (commonly 68% is used). It is calculated as

5 $$\begin{aligned} \text {BCEA} = 2k\pi \sigma _x\sigma _y\sqrt{1-\rho ^2} \end{aligned}$$

where $\sigma _x$ and $\sigma _y$ denote the standard deviation of the horizontal and vertical components of the gaze signal, $\rho$ the Pearson correlation between the horizontal and vertical components of the gaze signal, and *k* is a scaling constant computed as $k=-\log (1-P)$, where *P* is the fraction of the gaze data that are within the contours of the ellipse. As per Niehorster, Li, and Lappe (2017) and Niehorster et al. (2020d), further descriptions of the spatial spread of the gaze data contained in the BCEA ellipse, such as its anisotropy, orientation, and the radii of its minor and major axes, can be derived. The interested reader is referred to these publications.

The function bcea() (Listings 11–13) implements Eq. 5. The input arguments azi and ele are segments of gaze directions expressed as azimuth and elevation angles in degrees, and P is the fraction of gaze samples that is to be included within the contours of the BCEA ellipse. The output is the area of the BCEA ellipse in degrees-squared.

### Data loss

Loosely, data loss refers to the amount of data that the eye tracker failed to record. Data loss can be operationalized in various ways. One common operationalization of data loss uses the number of samples that are reported as invalid by the eye tracker (e.g., Holmqvist et al., 2012; Nyström et al., 2025a; Hooge et al., 2022). Invalid data (see Fig. 1B) is reported by different eye trackers in different ways, such as by special values ((0,0) or (-x_res,-y_res) where x_res and y_res are the resolution of the eye tracker screen), by reporting Not-a-Number (NaN), or by reporting no value at all (the sample is dropped). Data loss in the gaze position signal occurs if the eye tracker fails to determine the gaze position for one reason or another, e.g., because the participant looks outside the range of the eye tracker, due to closure of the eye, because the participant moved outside the eye tracker’s headbox, or because of hardware limitations. Data loss can also occur in other data reported by the eye tracker, such as pupil size. A single sample reported by the eye tracker may contain both valid and missing data, such as when the eye tracker is able to report eyelid separation but not pupil size because the eye is closed (Nyström et al., 2024), or a pupil size but not a gaze position. The researcher should decide whether the sample should be classified as entirely invalid or whether the valid portion of the data should be used in the analysis.
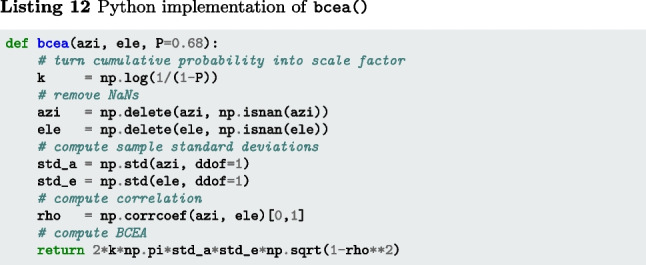

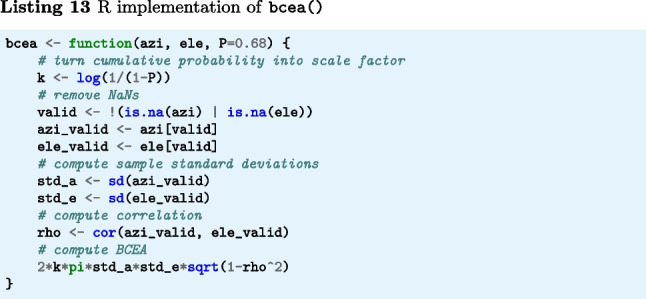


Data loss can have various durations. While sometimes larger sections of data are lost (e.g., during blinks, or because the participant looks away from the screen, see Fig. 1B), data loss may also occur in the form of short periods of data loss when tracking of the eye is unstable (e.g., Hessels et al., 2015a; Hessels et al., 2017, see Fig. 1B). Blinks may occur up to about 20 times per minute and typically last from about 150 ms to 400 ms (Stern, Walrath, & Goldstein, 1984; Nyström et al., 2024). As such, up to about 10 % of data loss can be expected purely due to blinks. In our experience, however, data loss in successful recordings is often substantially less.
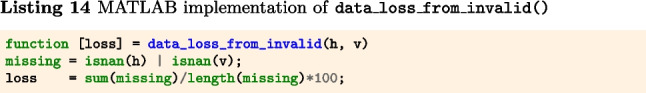

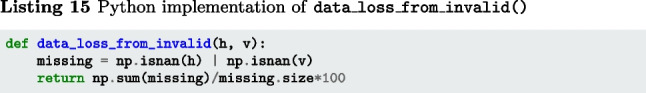

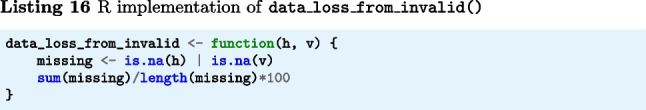


#### Fraction of invalid samples

The data loss in a signal can be operationalized in various ways. For instance, the total duration of lost samples can be summed to provide the data loss duration in seconds, an absolute measure of data loss. Often, however, a relative measure of data loss is preferred to indicate what percentage of a recording’s data was lost. One way to operationalize the percentage of data loss is as the ratio of invalid samples to all received samples.

6 $$\begin{aligned} \begin{aligned} \text {data loss}&= \frac{\text {number of invalid samples}}{\text {total number of samples}}*100\% \end{aligned} \end{aligned}$$

The function data_loss_from_invalid() (Listings 14–16) implements Eq. 6. Input arguments h and v are segments of horizontal and vertical gaze data. It does not matter what units or coordinate system the gaze data is represented in. It is expected that invalid samples in the input are marked as Not-a-Number (NaN). If invalid samples are represented otherwise in the eye-tracking data, they should be replaced by NaNs. The output of this function is a percentage of data loss.

#### Ratio of number of valid and expected samples

However, if the eye tracker sometimes reports samples at a reduced frequency or does not report samples at all in the case of data loss or due to other technical issues (see, e.g., Hessels et al., 2015b), this measure would underestimate the extent of data loss. Data loss can therefore also be operationalized as the ratio of the number of received valid samples to the expected number of samples given the nominal sampling rate of the eye tracker (i.e., the sampling rate advertised on its specification sheet):

7 $$\begin{aligned} \begin{aligned} \text {data loss}&= \Big (1-\frac{\text {number of valid samples}}{\text {number of expected samples}}\Big )*100\%\\&= \Big (1-\frac{\text {number of valid samples}}{\text {recording duration}*\text {nominal sampling frequency}}\Big )*100\% \end{aligned} \end{aligned}$$

The function data_loss_from_expected() (Listings 17–19) implements Eq. 7. Input arguments h and v are as for data_loss_from_invalid(), duration is the duration of the recording in seconds, and frequency the expected sampling frequency of the eye tracker in Hertz. The output of this function is a percentage of data loss.
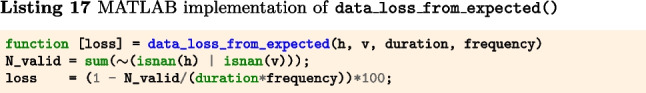

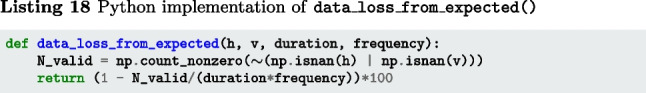

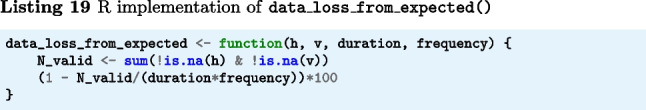


#### Effective frequency

However, some eye trackers may operate at a different frequency than advertised, a variable frequency (e.g., adjusted based on system load), or there may be no advertised sampling frequency at all. To handle such situations, Hooge et al. (2022) proposed calculating the effective sampling frequency of a recording, operationalized as

8 $$\begin{aligned} \begin{aligned} \text {effective frequency}&= \frac{\text {number of valid samples}}{\text {recording duration}} \end{aligned} \end{aligned}$$

Hooge et al. (2022) and Nyström et al. (2025a) argue that empirically estimated sampling frequency should be reported, “since samples sometimes may not be reported by the eye tracker and since some eye trackers have variable sampling frequencies (Hessels et al., 2015b; Liu, Zhao, Ren, Wang, & Zheng, 2018; Hooge et al., 2022). It is therefore important that researchers compute and report sampling frequency from data recorded in their own experiments.” (p. 16, Nyström et al., 2025a).

The function effective_frequency() (Listings 20–22) implements Eq. 8. Input arguments h and v are as for data_loss_from_invalid(), and duration is the duration of the recording in seconds. The output of this function is the effective frequency in Hertz.
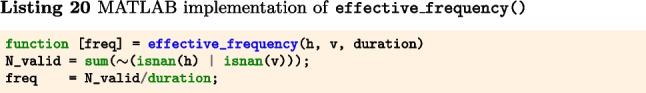

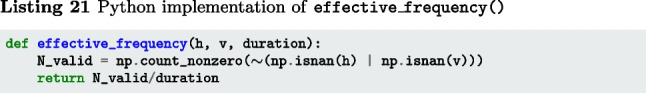

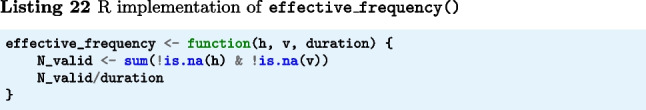


## How do I determine data quality?

As argued in the introduction, researchers should take an interest in the data quality of all their recordings. Simply taking values quoted on the manufacturer’s specification sheets at face value is not sufficient, and only determining data quality during a pilot, while important for study design (Hessels et al., 2025b), does not provide the needed information to judge the actual recorded data upon which the study’s conclusions rest. Indeed, Dunn et al. (2023) stipulate that empirically derived data quality should be reported for studies using eye trackers. As such, researchers need to ensure that data quality can be determined for each of their recordings. How does one determine eye-tracking data quality? Imprecision and data loss can generally be determined using the whole recording as precision only requires that participants fixate frequently, and data loss does not require any specific behavior from the participant. Determining inaccuracy, on the other hand, requires that specific procedures have been followed during the recording. Specifically, moments need to be built into the recording containing visual stimuli such as fixation targets that (often along with instructions) allow assuming with reasonable certainty where the participant looked (see the section “Inaccuracy” above for further discussion). Then, given a protocol that is meant to elicit such suitable gaze behavior, the offset between the assumed gaze position and the gaze position reported by the eye tracker can be determined.

In this section, we will delve into how one determines eye-tracking data quality in practice. To be able to determine the data quality of a recording, one needs:

1. Gaze data suitable for determining data quality (that is, as discussed above, gaze data recorded while a participant looks at known locations).
2. Software for computing eye tracking data quality from this gaze data.

In this section, we provide both protocols for collecting suitable eye-tracking data and tools for automated determination and reporting of data quality.

**Fig. 4:**
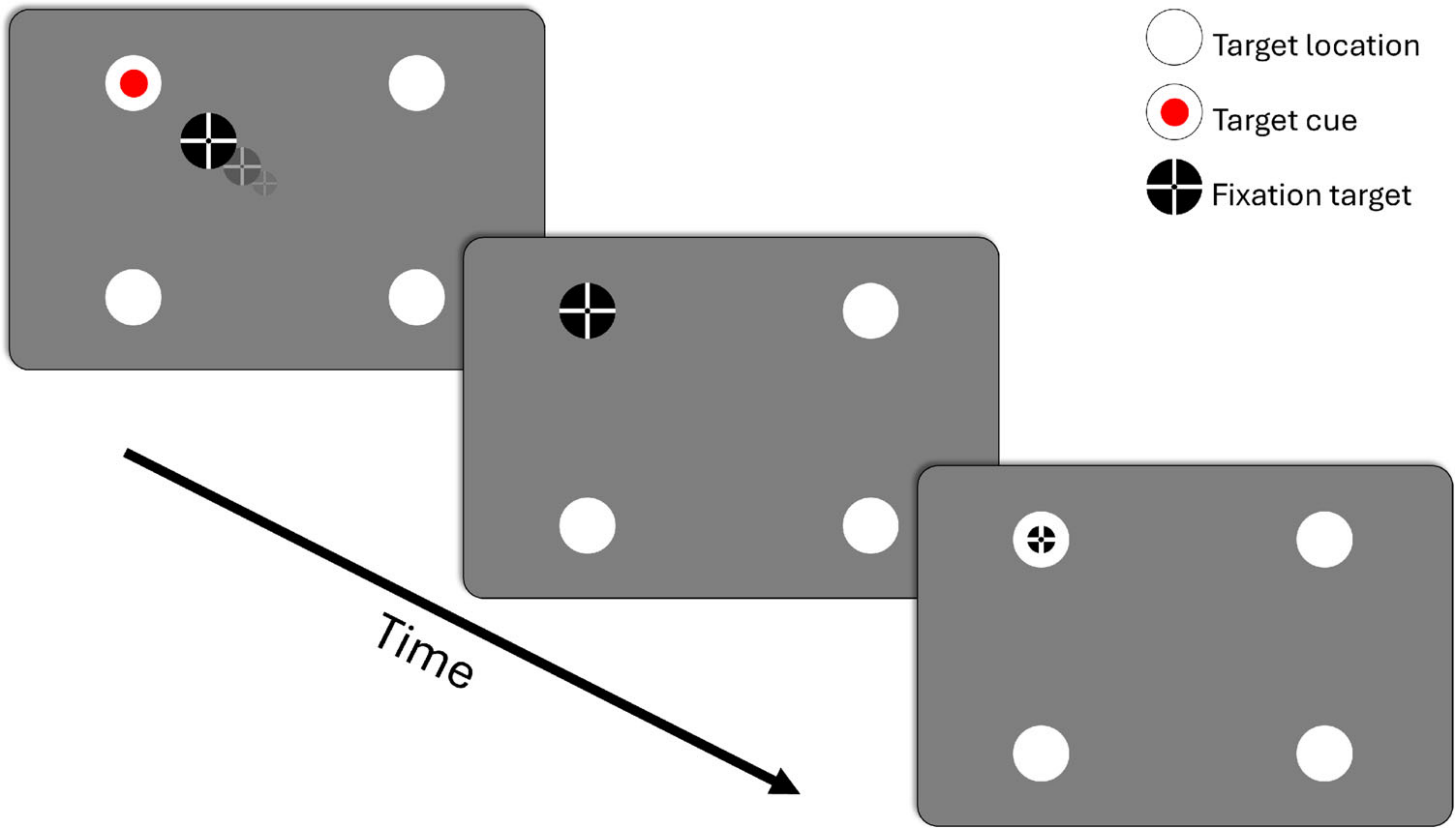
Accuracy assessment (validation) procedure. As an example, four fixation target locations are indicated by white circles. In the first panel, the fixation target (*black circle with a white cross* and a *smaller black circle* overlaid, Thaler, Schütz, Goodale, & Gegenfurtner, 2013, ABC; see also Niehorster et al., 2025c) is moving towards the fixation target location cued by the *red circle*. In the middle panel, the fixation target has arrived at the target location. In the last panel, the fixation target has shrunk to half its size, which is the size it has during the interval that is used for the accuracy determination. After validation data has been collected for the fixation location, the fixation target moves to the next location, and the same sequence shown in this sequence is repeated until data has been collected for all fixation target locations

While the discussion in the sections “Inaccuracy” and “Imprecision” above applies to both wearable and screen-based eye trackers, the procedures for determining inaccuracy and imprecision differ in practice between the two. A screen-based eye tracker is not only usually attached to a display on which a dynamic validation stimulus can be shown under the experimenter’s control, but also this type of eye tracker usually delivers gaze data directly in screen coordinates and synchronized to the screen. This makes performing a validation with a screen-based eye tracker and calculating eye-tracking data quality from the recording relatively straightforward. Wearable eye trackers, on the other hand, record eye movements with respect to the participant’s head and only provide a simultaneously recorded video from a front-facing scene camera as context (Nyström et al., 2025a; Lappi, 2016; Hessels, Niehorster, Nyström, Andersson, & Hooge, 2018; Hooge et al., 2024b). The data delivered by such an eye tracker is thus not referenced to any object in the world (such as fixation targets on a validation display or poster), nor temporally synchronized to a validation procedure (Niehorster et al., 2023; Niehorster, Hessels, Nyström, Benjamins, & Hooge, 2025a), which poses additional requirements for the data collection and requires a different analysis approach. As such, below we separately describe the approaches for performing an accuracy assessment for these two classes of eye trackers.

While the discussion of wearable eye trackers builds on existing tools, this section introduces a simple protocol and a new tool (ETDQualitizer) for determining data quality in screen-based eye trackers. Is a new tool needed for determining the data quality of screen-based eye trackers? It depends. If one’s academic work occurs within a single ecosystem (e.g., using an EyeLink eye tracker and its Experiment Builder software, or a Tobii eye tracker and the Tobii Pro Lab software), one likely already has a solution for determining data quality and may not be interested in what we present here. The tool presented here caters to those who are not in this position, for instance, because they use custom tools with their eye trackers or because their eye trackers do not provide such facilities at all. There are, however, other reasons why one may wish to use the eye tracker-agnostic and open workflow and tools we present and discuss here. First, the methods manufacturers use to determine data quality, such as the underlying calculations and procedures for sample selection, are often not transparent. This means manufacturers’ offerings may not be suitable if one wishes to control how data quality is determined, or at least have insight into the methods. Second, if one works across multiple eye tracking setups, e.g., in different labs or because one’s lab has acquired a new system, one may wish to use the same method for determining data quality to ensure comparability (cf. glassesValidator for wearable eye trackers, Niehorster et al., 2023).

### Screen-based eye trackers

#### Collecting gaze data suitable for accuracy assessment

We have developed a validation procedure to enable conducting accuracy assessments of screen-based eye-tracking recordings. This procedure is implemented in a PsychoPy script (available from https://github.com/dcnieho/ETDQualitizer/tree/master/python/ETDQualitizer/validator) that shows a configurable number of validation targets according to the following protocol. We will refer to this script as the *Validator script*. The validation procedure described here is illustrated in Fig. 4. First, the experimenter chooses the number, locations, and other properties of the fixation targets used during the validation. Depending on the experiment’s needs, only a single target in the center of the screen may be used, but often a grid of targets is used. Examples are a 3x3 grid (this is the default for the EyeLink), four points laid out in a diamond-shaped pattern (Niehorster et al., 2020a), or two rows of four targets each (Niehorster et al., 2024). The researcher may consider selecting a set of targets that span the expected range of gaze positions during their recording. The fixation targets are presented to the participant one at a time, in random order. A random presentation order is used to prevent participants from anticipating the next fixation target, reducing the chance that they look away too early from the currently presented fixation target. The fixation point is smoothly animated from one fixation location to the next to make it easy for participants to follow along. While the fixation target is in flight to its next location, the upcoming fixation target is cued by means of a red dot. When the fixation target arrives at the next target location, it shrinks to a smaller size and remains at that size for a given duration. Eye-tracking data for accuracy determination is collected during this period, when the fixation target is small. The period during which the target shrinks gives the participant time to establish fixation of the target location before the collection of validation data commences, increasing the chance that the participant accurately maintains fixation of the center of the fixation target during the interval used for accuracy determination.

It should be noted that this protocol is appropriate for participants who are able to follow instructions. For participants who cannot follow instructions, such as human infants and nonhuman primates, other procedures have been developed (e.g., Hessels & Hooge, 2019; Niehorster et al., 2024), while gamified versions for younger children have also been developed (e.g., Špakov et al., 2019).

The Validator script (i.e., the PsychoPy script implementing the above validation protocol) supports recording eye tracking data from Tobii, SMI, and SR Research (EyeLink) screen-based eye trackers. Support for other systems can be easily added by users with programming skills due to its modular design. The authors welcome the reader to contribute any such enhancements to our repository at https://github.com/dcnieho/ETDQualitizer. The Validator script saves the recorded gaze data in the input format expected by the ETDQualitizer tool described in the next section, which computes the various data-quality measures. This data format is a tab-separated file with a timestamp column, horizontal and vertical gaze position columns for one or two eyes, a target assignment column indicating what bit of the data should be used for calculating data quality for each target (this is when the validation target has shrunk to its smallest size, cf. the validation protocol and Fig. 4) and finally two columns indicating the horizontal and vertical target position during these intervals.

#### ETDQualitizer: Determining data quality for screen-based eye tracker recordings

In this section, we present ETDQualitizer. ETDQualitizer is a tool that enables anyone to easily determine the data quality of their recordings. A version that can be run directly in a web browser is available at https://dcnieho.github.io/ETDQualitizer. This web browser version can be used without installing anything, does not require a single line of programming code, and runs locally (no data is uploaded to the Internet). In addition to the web browser version, ETDQualitizer is available as a pip-installable Python package (pip install ETDQualitizer), an installable R package (install.packages("ETDQualitizer")) and a MATLAB package (ETDQualitizer) that can be installed from the Add-Ons manager. These packages include adaptations of the reference implementations for determining the various eye-tracking data quality measures (listings 1–22), and the conversion routines described in the appendix (listings 23–40). These functions can be called directly by the advanced user. For the user who does not want to deal with this complexity, ETDQualitizer also includes scripts that do all the heavy lifting and can use the data files produced by the Validator script described above to directly determine data quality for screen-based eye tracker recordings. The implementations for the three programming languages, as well as the web browser version, are equivalent and produce the same output for the same input eye-tracking data files.

The ETDQualitizer scripts take as input gaze position data in the format produced by the Validator script described above. Such data can be recorded directly by this PsychoPy script, but the scripts can also be run on gaze data collected in any other way, as long as the data is formatted in the same way. For a given data file, the function compute_data_quality_from_validation() provides as output a table with all the data quality measures computed per eye and fixation target. The ETDQualitizer library also includes a function, report_data_quality_table(), that from a set of these output tables produces summary statistics across participants, and that can generate a text reporting on the eye-tracking data quality of a set of recordings that can be directly pasted in a scientific report about the study (see the section ”How should I report data quality?” below).

#### Using the Validator script and ETDQualitizer

This section provides a step-by-step procedure for using the PsychoPy Validator script and ETDQualitizer to determine data quality for the recordings of a screen-based eye tracking study. Note that steps 3–5 in the procedure below can also be performed with the web version of ETDQualitizer, in which case no programming, or calling of the indicated functions is required. Configuration of the validator script, further usage details of the ETDQualitizer tool, and a complete walkthrough of its functionality are provided in the readme at https://github.com/dcnieho/ETDQualitizer.

1. *Preparation to collect eye tracking data using the Validator.* Before recording, the researcher prepares a recording protocol. This protocol may include one or multiple runs of the standalone Validator script available from https://github.com/dcnieho/ETDQualitizer/tree/master/python/ETDQualitizer/validator. It is, however, also possible to call the function validator.run_validation() from an existing PsychoPy script at the appropriate time(s). This function implements the actual validation routine and allows validation to be added to existing PsychoPy scripts with just a single line.
2. *Recording of eye tracking data.* During recording, the participant views the validation screen. The participant is given the following instructions for completing the validation procedure: *A fixation target will be shown and will move to different positions on the screen. When the target pauses at a location on the screen, carefully fixate on its center and do not blink. Keep your gaze steady on the center of the target for the entire time the target remains at that location. When the target moves again, follow it to the next location.* Each run of the validation script produces a data file that is the input to the next step.
3. *Analysis.* To determine data quality, the data files produced by the PsychoPy validation script are loaded and provided to ETDQualitizer’s compute_data_quality_from_validation() function one at a time. This function provides as output a table with the various eye-tracking data quality measures calculated for the validation session, per eye and fixation target. Example scripts that show how to use this function, and example input data as recorded with the PsychoPy script, are provided at https://github.com/dcnieho/ETDQualitizer/tree/master/example.
4. *Result summary.* The eye-tracking data quality measures determined for each session (output from step 3) can then be summarized into a single average per data quality measure (along with accompanying standard deviation and range of values) using the function report_data_quality_table().
5. *Reporting.* The function report_data_quality_table() provides as output also a text including the calculated data quality measures that can be directly pasted into a scientific report. Example texts generated by this function are shown in the “How should I report data quality?” section below.

### Wearable eye trackers

As discussed above, assessing the data quality of wearable eye-tracking recordings poses distinct issues that are addressed through distinct procedures and analysis tools. The paper about the glassesValidator tool we have previously developed (Niehorster et al., 2023; also integrated in gazeMapper, Niehorster et al., 2025a) provides a detailed discussion to which the interested reader is referred. Here, we briefly discuss how data quality is assessed using this tool.

First, as with screen-based eye trackers, a set of fixation targets is required that are easily presented to participants and that are designed so that it is easy to instruct participants to fixate them. To this end, we use a poster (see Fig. 2 in Niehorster et al., 2023) on which several fixation targets are printed. Furthermore, a way for a computer program to locate the poster in the scene video of the wearable eye tracker is needed. This is accomplished by placing fiducial markers on the poster in the form of specially designed 2D barcodes called ArUco markers (Garrido-Jurado, Munoz-Salinas, Madrid-Cuevas, & Medina-Carnicer, 2016). Once the poster is detected, gaze can be referenced to the poster, and thus the distance on the poster between a gaze position and a given fixation target can be determined. Finally, a way of knowing *when* a participant is looking at the fixation targets is needed. When using glassesValidator, this is solved by the experimenter hand-coding when in the recording an accuracy assessment (validation) episode takes place. The rest of the processing of a recording then takes place automatically using glassesValidator, and the output provided by glassesValidator is the same data quality metrics as ETDQualitizer provides for screen-based eye tracker recordings.

### Uses of eye-tracking data quality values

Once one has determined the data quality for a given eye tracking recording, how can one use the inaccuracy, imprecision and data loss values? Besides reporting the values, they have several other uses. For instance, in a gaze-contingent study, they can be used to describe the stimuli presented to participants (e.g., Niehorster, Cornelissen, Holmqvist, & Hooge, 2019). The values can also be used for exclusion of data that does not meet the required level of data quality. This can be done either based on a priori standards for what constitutes sufficient data quality, or based on statistical criteria for removing outliers. For instance, if one conducts a reading study or a visual search study, one can determine beforehand what level of inaccuracy one can permit when performing analysis using a given set of AOIs (Hooge et al., 2026; Orquin & Holmqvist, 2018). Data with worse inaccuracy than the predetermined acceptable level can then be excluded. Similarly, levels of imprecision and data loss can be used to exclude recordings of insufficient quality (e.g., Hessels et al., 2023). Alternatively, a statistical criterion such as inaccuracy, imprecision or data loss exceeding the mean + 2 standard deviations across the participant population can be used as exclusion criterion.

## How should I report data quality?

Now that the eye-tracking data quality for a set of recordings has been determined, it needs to be reported. How does one do so? Here we provide a brief discussion, building on the guidelines of Dunn et al. (2023, points A9, A10, and A12). Before we provide these examples, we first want to emphasize again that the reported eye-tracking data quality should be based on assessments performed as part of all recordings included in the analysis, not based on only a subset or on some pilot recordings. Furthermore, when reporting eye-tracking data quality, not only should numerical values be provided, but it should also be described how these values were derived. An example of how this required context could be provided when reporting data quality is: “For 7 participants, the average [*data quality measure*] of the gaze data, determined from a 9-point validation procedure using ETDQualitizer [*version number*] (citation to the current paper), was ...”.

### Accuracy

For any study analyzing looking behavior with respect to a given direction, the accuracy of the eye-tracking recordings should be reported. Different from what was written in Dunn et al. (2023), we emphasize here that accuracy should be reported regardless of whether the eye tracker requires calibration or not. For instance, some remote eye trackers (e.g. various Tobii eye trackers) and wearable eye trackers (e.g., the Pupil Labs Neon and Invisible and the Tobii Glasses X) do not require calibration, whereas other systems such as the SeeTrue STONE only require calibration once for a participant instead of once per recording. In all these cases, the accuracy, as for instance derived using a validation procedure, of all recordings included in the study should nonetheless be reported. An example of how to report accuracy (produced by the report_data_quality_table() function in ETDQualitizer) is the following: “the average inaccuracy of the gaze data [...] was 0.80$^{\circ }$ (STD 0.28$^{\circ }$, range 0.46$^{\circ }$–1.11$^{\circ }$)”.

### Precision

As Dunn et al. (2023) state, “For any quantities reported, the estimated uncertainties should be stated”. RMS-S2S and STD are two measures commonly used for this purpose, although depending on the study context, BCEA may, for instance, also be relevant to report. An example of how to report precision (produced by the report_data_quality_table() function in ETDQualitizer) is the following: “Average RMS-S2S precision was 0.084$^{\circ }$ (STD 0.036$^{\circ }$, range 0.057$^{\circ }$–0.161$^{\circ }$) and STD precision 0.184$^{\circ }$ (STD 0.214$^{\circ }$, range 0.088$^{\circ }$–0.668$^{\circ }$)”.

### Data loss

Data loss typically refers to a percentage of lost samples in a recording. Depending on whether the eye tracker has a fixed sampling frequency and whether it reports invalid samples when track is lost (Hessels et al., 2015b), one of the various data loss metrics may be pertinent to report. As Dunn et al. (2023) state, when the eye tracker has a fixed sampling frequency, data loss may be reported as a percentage of lost samples. We wish to emphasize that whether it is appropriate to operationalize data loss using simply the total number of samples or the expected number of samples (see the section “Data loss” for a discussion) depends on whether the eye tracker reports lost samples at the same rate as valid samples. If the eye tracker does not have a fixed sampling frequency, the reader should consider reporting the effective sampling frequency (Hooge et al., 2022). Example reports of data loss are: “Across 4 participants, the average data loss was 3% (STD 4%, range 0%–13.4%)” and “The average effective frequency (Hooge et al., 2022) was 582 Hz (STD 24 Hz, range 520 Hz–600 Hz)”.

### Further considerations

The goal when reporting eye-tracking data quality in a paper is not to strive for completeness, but to provide the measures that are relevant for judging the appropriateness of the analyses and the degree of confidence one should have in them and the derived conclusions. As such, this discussion regarding which eye-tracking data quality measures to report and how to report them should not be seen as a license for the reader to stop exercising their independent judgment. There are various other eye-tracking data quality aspects or eye tracker specifications that may provide important context to one’s analyses or conclusions (see, e.g., McConkie, 1981; Holmqvist et al., 2011; Holmqvist et al., 2012; Niehorster et al., 2020c; Niehorster et al., 2020d; Nyström et al., 2025a; Jakobi et al., 2024, for discussions) but that are not captured in the discussed accuracy, precision and data loss measures. If appropriate, one should go beyond the measures discussed in this paper and report also any other measures that are of relevance to the study. Examples are provided by Choe et al. (2016, their Figure 2c), who report systematic drift (changing inaccuracy) of the gaze position signal during the course of their recordings, Aguilar and Castet (2011) and Saunders and Woods (2014) who measure the latency of gaze-contingent display systems, and Hessels et al. (2025a) who report the latency between the gaze data and scene camera of the wearable eye trackers in their study.

A further important consideration is that one may want to split out the reported eye-tracking data quality measures between conditions or between participant groups. As discussed in the introduction, there may be large differences in aspects of eye-tracking data quality, such as imprecision and data loss, for instance, between cohorts of children of different ages (Hessels & Hooge, 2019). These differences may affect the output of the methods used for analyzing the eye tracking data. In such cases, it would be pertinent for the authors of the study to report data quality separately for each participant group. A similar consideration applies in within-subject designs, where each participant is exposed to multiple experimental conditions. If it is expected that data quality may differ between conditions, e.g., because participants move more in one condition than another, or if a pilot showed this to be the case, reporting data quality per condition may be pertinent. Being able to do so may require specific adaptations to the recording procedure to enable determining data quality for each condition, instead of only once at the beginning of the recording.

## Concluding remarks

We hope that, through this paper, we have both convinced the reader of the importance of taking a data quality perspective during the design, analysis, and reporting of their study, and equipped them with the tools required to understand, determine, and report eye-tracking data quality. We think that this will both improve the quality of studies performed with eye trackers and the usefulness of the reports written about these studies to other researchers, as adequate context is provided to appreciate the reported findings. As such, while we have seen that reporting practices in eye-tracking studies have improved over the last decade, we hope that efforts such as this paper and others (Dunn et al., 2023) continue to raise the quality of eye-tracking research.

## Figures and Tables

**Fig. 5:**
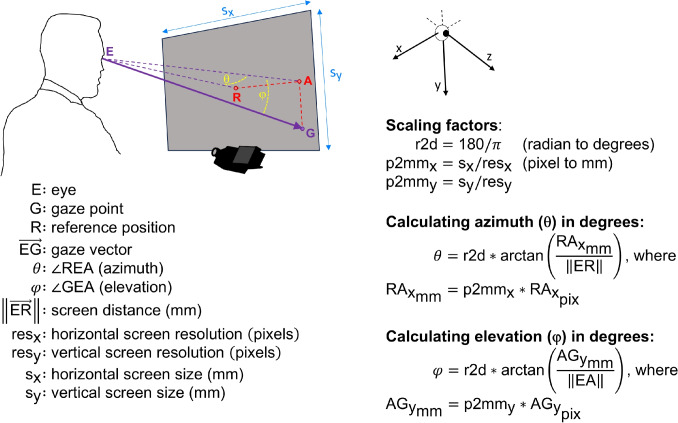
Gaze expressed in different coordinate systems. The image shows a gaze point (**G**, *purple point*) on a screen and associated gaze vector $\overrightarrow{EG}$ (*purple arrow*). This gaze point **G**, expressed in pixel coordinates with respect to a reference position on the screen (**R**, *red point*, here the center of the screen), is first converted to mm. Note that **R** is the point on the screen where the vector $\overrightarrow{ER}$ (*purple dotted line*) is perpendicular to the screen. Then, together with the screen distance ($\Vert ER\Vert$), the gaze position is transformed into a gaze direction expressed in a spherical coordinate system as azimuth ($\theta$) and elevation ($\varphi$) angles in Fick (1854) order. Cartesian coordinates are expressed with the origin at the center of the eye. The positive *Z*-axis points forward to the reference position. The positive *X*-axis points rightward and the positive *Y*-axis downward

**Fig. 6:**
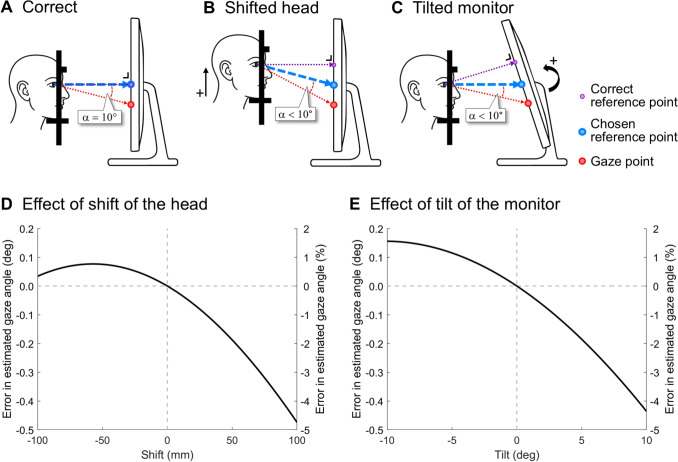
Impact of choosing the wrong reference point. **A** The reference point (*blue dot*, here the center of the screen) is chosen correctly when it coincides with the point on the screen (*purple dot*) where the vector from the eye (*purple dotted line*) is perpendicular to the screen. Errors in the setup geometry may cause the vector from the eye to the chosen reference point to be nonperpendicular to the screen. Such errors can be expressed in at least two ways, i.e., that the head is shifted with respect to the screen (panel **B**) or that the screen is tilted (panel **C**). Note that in panels B and C, the vector to the chosen reference point (*blue arrow*) is no longer perpendicular to the screen, and that the chosen reference point does not correspond to the point (*purple dot*) where the vector from the eye is perpendicular to the screen. If the reference point is nonetheless incorrectly placed at the center of the screen in these situations, the gaze angle to a point on the screen would be over- or underestimated by the procedures presented in this appendix. To examine the under- and overestimation, we placed a point (*red dot* in panel A) at a gaze angle of ${10}\,{^{\circ }}$ below a correctly chosen reference point (denoted by $\alpha$) on a screen placed at a viewing distance of 65 cm. We then simulated a shift of the observer’s head (panel B) or a tilt of the screen (panel C), due to which the actual gaze angle with respect to the chosen reference point that is required to look at the gaze point no longer equals $\alpha$, despite the gaze point being at an identical position on the screen. Panels D and E quantify the resulting errors. **D** When the head is shifted, errors range from a small overestimation of the gaze angle for small downward shifts (up to about 0.1 $^{\circ }$, 1 %) to up to about 0.5 $^{\circ }$ (5 %) underestimation for a significant upward shift of 100 mm. **E** When the screen is tilted backward, a small overestimation of the gaze angle (up to about 0.2 $^{\circ }$, 2 %) results, while tilting the screen in the other direction leads to underestimation of up to about 0.4 $^{\circ }$ (4 %) for a 10 $^{\circ }$ tilt

## Data Availability

Code available at https://github.com/dcnieho/ETDQualitizer.

## A: Expressing gaze data as positions, angles, or vectors

Gaze data can be expressed in different coordinate systems, different reference frames and using different units. Outputs commonly provided by eye trackers include, for instance, pixel coordinates on a computer screen for screen-based eye trackers and pixel coordinates on the scene camera image for wearable eye trackers. Some eye trackers may instead, or additionally, provide 3D vectors representing the gaze direction. Gaze measures are, however, commonly expressed as angles in degrees, since such a representation is easy to understand, physiologically meaningful, and allows for direct comparison between studies. In contrast, gaze vectors consist of three values whose interpretation is not intuitive, and positions on a screen require additional context such as screen size, screen resolution, and viewing distance for interpretation (e.g., 100 pixels on a smartphone screen held at a 30-cm distance are very different than 100 pixels on a 24-inch computer screen placed at 80 cm).

Imagine you need to report the average inaccuracy and imprecision of your recordings and to follow standard and recommended practice (Dunn et al., 2023) you want to report these values as angles in degrees. The eye tracker, however, provided a file with gaze positions in pixels on the screen. How do you work with this gaze data and convert it into an angular representation? This supplementary section discusses procedures for converting gaze represented as screen positions or gaze vectors to angular representations, as well as converting angles back to screen positions or gaze vectors. Familiarity with these procedures and associated terms is not only required to be able to process the gaze signal that an eye tracker delivers, for instance, to determine the data quality of eye-tracking recordings, but also helps understanding what an inaccuracy of, for instance, 1.8 $^{\circ }$ means in practical terms. Importantly, converting gaze data to an angular representation is also required to follow the descriptions and computer code in the main part of the article, since it is assumed that gaze is represented as a pair of horizontal and vertical angles. In this section, we not only provide a discussion, but also mathematical and algorithmic (in  ,  , and  ) representations of these conversion operations.

### A.1: From position to angle

How can one transform the eye tracker signal (expressed as a screen position in pixels) into gaze angles in degrees? Consider Fig. 5, which depicts a participant looking at a screen. The eye tracker reports that the participant is looking at the gaze point **G**, expressed in pixel coordinates in the screen’s reference frame. To transform this gaze position to gaze angles, a reference position (**R** in Fig. 5) with respect to which these gaze angles are expressed must be chosen. The procedures presented in this section are only valid under the condition that the axis from the observer’s eye to the chosen reference position on the screen ($\overrightarrow{ER}$ in Fig. 5) is perpendicular to the screen. Choosing the correct reference position given the geometry of one’s setup, or adjusting the setup such that the desired reference point is at the same location as the correct reference point, is thus critical to prevent violating the above assumption. Some may simply choose the center of the screen as the reference point, irrespective of the geometry of their setup. In this case, the procedures below will produce slightly incorrect gaze angles. More insight into the introduced error is provided in the section “Impact of choosing the wrong reference point” below. Various procedures for deriving head-relative gaze orientation for wearable eye trackers from gaze positions expressed as coordinates in the eye tracker’s scene video are discussed in detail elsewhere (Hooge et al., 2022, 2024b; Niehorster et al., 2023, 2025a) and will not be further discussed here. If the wearable eye tracker provides gaze vectors, procedures for converting them to gaze angles are provided below in the “From vector to angle” section.

To be able to express gaze as angles away from the reference position on the screen, the gaze point provided in pixels by the eye tracker first has to be converted to physical positions on the screen, for instance, expressed in mm. To do so, one needs to know the size of the screen (specifically, the display area, that is, the light-emitting part of the screen) in mm and its resolution in pixels. Given the horizontal size of the screen ($\text {s}_x$) and the horizontal resolution of the screen ($\text {res}_x$), a scaling factor to convert between pixels and mm can be calculated:

9 $$\begin{aligned} \text {p2mm}_x = \frac{\text {s}_x}{\text {res}_x}, \end{aligned}$$

and similarly a scaling factor $\text {p2mm}_y$ can be determined from the vertical size of the screen ($\text {s}_y$) and the vertical resolution of the screen ($\text {res}_y$)^4^. In code, converting positions on the screen in pixels to positions in mm using the p2mm factors looks as shown in Listings 23–25. Note that the $\text {p2mm}_x$ and $\text {p2mm}_y$ scaling factors are computed in these functions and directly applied to the input data in pixels (x and y) to return gaze position data expressed in mm.

Once physical positions (expressed, e.g., in mm) on the screen are known, these can be used to determine physical distances on the screen. This is done by subtracting a reference position (e.g., **R** in Fig. 5) from the reported gaze position (**G**)^5^. Let us unpack this into elementary scalar subtractions. To determine the distance between the horizontal component of the gaze position and the reference position on the screen, one subtracts the horizontal coordinate of **R** from the horizontal coordinate of **A**, i.e., $RA_x=A_x-R_x$ (or equivalently using points **R** and **G**, $RG_x=G_x-R_x$). Similarly, the vertical component can be calculated using points **A** and **G**, i.e., $AG_y=G_y-A_y$ (or equivalently using **R** and **G**, $RG_y=G_y-R_y$)^6^.

Given these distances on the screen and the viewing distance to the screen (all expressed in mm), the angular gaze direction in degrees can then be calculated by the following two equations:

10 $$\begin{aligned} \begin{aligned} \theta&= \text {r2d}*\text {atan2}(RA_x,\Vert ER\Vert )\\ \varphi&= \text {r2d}*\text {atan2}(AG_y,\Vert EA\Vert ), \end{aligned} \end{aligned}$$

where $\Vert ER\Vert$ is the distance between the eye and the screen (putting two points such as *ER* between the vertical pipes symbol $\Vert$ denotes calculating the absolute distance between these points), $\Vert EA\Vert$ the viewing distance to point **A** on the screen ($\Vert EA\Vert =\sqrt{\Vert RA\Vert ^2+\Vert ER\Vert ^2}$), $\text {atan2}()$ the two-argument (signed) version of the inverse of the tangent function, and $\text {r2d}$ a scaling factor to convert radian to degrees ($180/\pi$). The resulting angular gaze direction is described as two angles using the Fick (1854) order^7^, called the azimuth ($\theta$ in the equations) and elevation ($\varphi$ in the equations). $\varphi$ performs the vertical rotation to bring gaze from gaze point **G** to point **A** on the screen, and $\theta$ then performs the horizontal rotation back to the reference position **R**.

Implemented in code, the conversion from positions to gaze angles looks as provided in Listings 26–28, where x and y are the horizontal and vertical components of the distance on the screen between the gaze position (**G**) and the reference position (**R**) and viewing_distance should be in the same units as x and y (e.g., mm).

### A.2: From angle to position

The reverse conversion, computing a gaze position on the screen from azimuth and elevation angles, can also be performed. The following equations transform a gaze position expressed as azimuth ($\theta$) and elevation ($\varphi$) angles in Fick (1854) order, along with the distance between the screen and the eye ($\Vert ER\Vert$ in Fig. 5), into a distance on the screen away from the reference position (e.g., in mm):

11 $$\begin{aligned} \begin{aligned} RA_x&= \Vert ER\Vert *\tan (\theta *\text {d2r})\\ AG_y&= \Vert ER\Vert *\frac{\tan (\varphi *\text {d2r})}{\cos (\theta *\text {d2r})}, \end{aligned} \end{aligned}$$

where $\tan ()$ is the tangent function, $\cos ()$ is the cosine function, and $\text {d2r}$ a scaling factor to convert degrees to radian ($\pi /180$). Implemented in code, this transformation looks as provided in Listings 29–31. To then turn a gaze position on the screen expressed as a distance from the reference position in mm into a position expressed in pixels, we have to first turn the distance from the reference position into a position on the screen: $G_x = RA_x + R_x$ and $G_y = AG_y + R_y$. To convert this position in mm to a position on the screen in pixels, the scaling factor to convert between pixels and mm from above (9) can be used. In code, converting positions on the screen in mm to positions in pixels using the p2mm factor looks as provided in Listings 32–34. Note that the $\text {p2mm}_x$ and $\text {p2mm}_y$ factors are computed in these functions and directly applied to the input data in mm (x and y) to return gaze position data expressed in pixels.

### A.3: From angle to vector

Some calculations require gaze to be expressed as a gaze vector instead of as two angles. This is, for instance, used internally in the code for computing inaccuracy (Listings 1–3 above). A gaze vector is a 3D set of values in Euclidean space that represents the direction of gaze, and often also the distance between the eye (**E** in Fig. 5) and the gaze point (**G** in Fig. 5). The following equations turn gaze expressed as azimuth ($\theta$) and elevation ($\varphi$) angles in Fick (1854) order, along with a viewing distance ($\rho$, $\Vert EG\Vert$ in Fig. 5), into a gaze vector:

12 $$\begin{aligned} \begin{aligned} x&= \rho *\cos (\varphi )*\sin (\theta )\\ y&= \rho *\sin (\theta )\\ z&= \rho *\cos (\varphi )*\cos (\theta )\\ \end{aligned} \end{aligned}$$

Often, only the direction but not the length of the vector is important for the subsequent calculations. In this case, $\rho$ can be set to 1, or omitted altogether. The code in Listings 35–37 implements this transformation.

### A.4: From vector to angle

Finally, some eye trackers deliver gaze vectors (for instance, many head-worn eye trackers, but also remote eye trackers such as those manufactured by Tobii and SMI). To transform these into azimuth ($\theta$) and elevation ($\varphi$) angles in Fick (1854) order, a slight adaptation of Equation 10 can be used. The following equations transform a gaze vector $\overrightarrow{EG}$ (expressed as *x*, *y* and *z* components in 3D space) into azimuth and elevation angles:

13 $$\begin{aligned} \begin{aligned} \theta&= \text {r2d}*\text {arctan}\Big (\frac{x}{z}\Big )\\ \varphi&= \text {r2d}*\text {arctan}\Big (\frac{y}{\sqrt{x^2+z^2}}\Big ), \end{aligned} \end{aligned}$$

where $\text {r2d}$ is a scaling factor to convert radian to degrees ($180/\pi$).

The code in Listings 38–40 implements this transformation.

### A.5: Impact of choosing the wrong reference point

As noted above, the procedures described in the sections “From position to angle” and “From angle to position” are only valid under the assumption that the axis from the observer’s eye to the reference position on the screen (the position with respect to which gaze angles are expressed) is perpendicular to the screen (see Fig. 6A). However, in a real measurement setup, it is possible that not everything is positioned and oriented perfectly. In such situations, it is easy to choose a wrong reference point that does not conform to the above assumption. Indeed, many researchers, including the authors of this article, routinely use the presented procedures to calculate gaze angles with respect to the center of the screen, even when not everything in their setup is perfectly placed or oriented perpendicularly. Is that problematic? To investigate this, we simulate the effect on the estimated gaze angle when small errors in orientation or positioning sneak into the setup.

To this end, we performed simulations (available from https://github.com/dcnieho/ETDQualitizer/tree/master/simulation) in which the axis from the observer’s eye to the reference position on the screen is not perpendicular. Errors in the geometry of a setup due to which the axis is not perpendicular can be described in at least two ways:

1. The observer’s head is shifted vertically or horizontally with respect to the screen (Fig. 6B). Such a shift would entail that the axis from the eye to the chosen reference position (here, wrongly, the center of the screen is chosen) is not perpendicular to the screen.
2. The screen is tilted (Fig. 6C), due to which the axis from the observer’s eyes to the chosen reference position on the screen (here the center of the screen) is not perpendicular to the screen.

In this simulation, we examined the effect of these two mistakes in the setup. We first determined a fixed point on a screen placed at 65-cm viewing distance (a common distance in eye tracking setups). This fixed point was chosen such that it is located at an angle of 10 $^{\circ }$ downward with respect to the axis from the eye to the reference position in a setup where this axis is perpendicular to the screen (as in Fig. 6A). We will refer to this fixed point as the gaze point. We then determined what the required actual gaze angle between the reference point and the gaze point is when the head is shifted (Fig. 6B) or the screen is tilted (Fig. 6C). Shifts of up to 100 mm and tilts of up to 10 $^{\circ }$ are used in this simulation, which likely exceed the range commonly observed in setups. Interested readers can use the simulation code at the link above to examine the effects of larger shifts or tilts.

The error in estimated gaze angle due to head shift is shown in Fig. 6D. Positive errors mean that the actual gaze angle is larger than the gaze angle that would be calculated when ignoring that the head is shifted, i.e., the gaze angle is underestimated. Negative values indicate that the actual gaze angle is smaller and thus that ignoring the imperfections in the setup’s geometry leads to overestimating the gaze angle. As can be seen in Fig. 6D, such underestimation only reaches about 5 % (0.5 $^{\circ }$ for the 10 $^{\circ }$ gaze angle used in this simulation) when the head is shifted up by 100 mm. For this downward gaze angle, if the head is instead shifted downward by up to 100 mm, an overestimation is observed of less than 1 % (0.1 $^{\circ }$). When the screen is instead tilted (Fig. 6E), the error ranges from an almost 2 % (0.2 $^{\circ }$) overestimation to an about 4 % (0.4 $^{\circ }$) underestimation for screen tilts of up to 10 $^{\circ }$. For context, a 1 $^{\circ }$ error corresponds to a distance on the screen of about 1.1 cm at the viewing distance used in this simulation.

In conclusion, for many applications, it is likely reasonable to use the calculations presented in this Appendix even when the setup geometry does not perfectly conform to the perpendicular viewing-axis assumption of these procedures. It should furthermore be noted that the errors reported here matter even less when comparing gaze angles between conditions or participant groups if the screen position and orientation with respect to the eye are similar across these comparisons.

## Footnotes

1 There are various other texts that deal with accuracy, precision and data loss, as well as other aspects of the data quality of eye tracker recordings and other eye tracker specifications (e.g., McConkie, 1981; Holmqvist et al., 2012; Holmqvist et al., 2011, Chapter 2.6; Niehorster et al., 2020c; Niehorster, Zemblys, Beelders, & Holmqvist, 2020d; Nyström et al., 2025a; Jakobi, Krakowczyk, & Jäger, 2024). An interested reader looking to delve deeper into these topics may be well-served by reading these texts.

2 Calibration is used to determine the mapping function that enables an eye tracker to provide meaningful output, such as the gaze position on a screen or eye orientation. Calibration often involves a procedure where one or multiple gaze targets are fixated. In contrast to calibration, validation does not establish the mapping function but evaluates its quality by assessing the accuracy of the calibrated gaze data. Note that not all eye trackers require a calibration (e.g., the Pupil Labs Neon, see Nyström et al., 2025a, for a discussion), but that validations can nonetheless also be carried out for such eye trackers. Note also that “calibration” is technically a misnomer; instead, “adjustment” would be the appropriate term for this action (BIPM et al., 2012, p. 28).

3 Note that for Python code in this paper, it is assumed that the import statement import numpy as np has been executed.

## Declarations

### Open Practices Statement

There is no data associated with the present article. Code available at https://github.com/dcnieho/ETDQualitizer.
